# Variation in digestibility parameters related to feed efficiency between and within two laying hen lines^☆^

**DOI:** 10.1016/j.psj.2025.105719

**Published:** 2025-08-23

**Authors:** Ghyslaine C.B. Schopen, Marco C.A.M. Bink, Estella Leentfaar, Dirkjan Schokker, Malou van der Sluis, Leon H. de Jonge, Lisanne M.G. Verschuren, Carmen Jansen-Noordijk, Esther D. Ellen

**Affiliations:** a Animal Breeding and Genomics, Wageningen University and Research, PO Box 338, 6700 AH Wageningen, The Netherlands; b Hendrix Genetics BV, Spoorstraat 69, 5831 CK Boxmeer, The Netherlands; c Institut de Sélection Animale B.V., Spoorstraat 69, 5831 CK Boxmeer, The Netherlands; d Wageningen Bioveterinary Research, PO Box 65, 8200 AB Lelystad, The Netherlands; e Animal Nutrition Group, Wageningen University and Research, P.O. Box 338, 6700 AH Wageningen, The Netherlands; f Topigs Norsvin Center B.V., PO Box 43, 6640 AA Beuningen, The Netherlands; g Current address: KWPN Royal Dutch Sport Horse, De Beek 109, 3852 PL Ermelo, The Netherlands

**Keywords:** Chemical analysis, Laying hens, Digestibility, Feed efficiency

## Abstract

To meet the increasing demand for eggs, while limiting the environmental impact, an increased efficiency of laying hen production is required. Feed efficiency (**FE**) plays a major role in this. In broilers, relationships between nutrient digestibility and FE have been observed, suggesting that information on digestibility coefficients (**DCs**) has potential to aid in prediction of FE. This study examines whether DCs, as determined by chemical analysis of laying hen manure, can serve as a predictor of FE in laying hens. In total, 100 laying hens from two lines differing in FE (line A and B) were studied. The objective of this study was two-fold: to investigate 1) the variation in DCs between and within the two lines, and 2) the use of DCs for prediction of FE in laying hens. Differences in performance and DCs were observed between the two laying hen lines. Line A hens showed a higher daily feed consumption and daily body weight, as well as lower egg mass, daily manure weights and laying percentages. Furthermore, all DCs were higher in line A than in line B. For line A, phenotypic correlations between DCs and feed conversion ratio (**FCR**) were moderate and ranged from 0.31 (DC for fat hydrochloric) to 0.60 (DC for organic matter). Correlations between residual feed consumption (**RFC**) and DCs ranged from 0.32 (DC for fat hydrochloric) to 0.79 (DC for dry matter). For line B, correlations between FCR and the DCs were not statistically significant, while the correlations between RFC and DCs ranged from 0.31 (DC for fat hydrochloric) to 0.43 (DC for organic matter). The contribution of different DCs in prediction models for FE differed between the two lines, making across-line FE prediction challenging in laying hens. Overall, the data on DCs collected in this study contribute to an improved understanding of differences in digestibility between laying hen lines and show potential for FE prediction.

## Introduction

Worldwide, there are approximately 7.5 billion laying hens, that together produce over 80 million tons of eggs per year (FAOstat, 2021), and the global egg production and consumption is expected to continue to increase (OECD/FAO, 2020). However, there is limited suitable land available for increased food production, due to, for example soil degradation (Gomiero, 2016). An increased efficiency of production is required, and feed efficiency (**FE**, defined as the ratio of production weight (in this case: egg mass) to feed intake; Carré et al., 2008) plays a major role in this production efficiency.

To increase laying hen FE, diets are formulated that match the hens’ nutrient requirements (Bryden et al., 2021). However, not all feed ingredients are fully digested by livestock (Nielsen et al., 2007) and birds have a compact gastrointestinal tract, which means that they need high quality and easily digestible feed (Wood and Willems, 2014). Digestive efficiency (that is, the proportion of dietary intake minus feces) can be difficult to determine in poultry due to mixture of feces and urine, and therefore determination of metabolizable efficiency (that is, the proportion of dietary intake minus feces and urine) is more practical (Vohra, 1972). Individual variation in nitrogen-corrected apparent metabolizable energy (**AMEn**) has been observed in broilers when they were fed wheat-based diets (Carré et al., 2002). Furthermore, AMEn has been observed to be heritable in broilers (Mignon-Grasteau et al., 2004). Moreover, it has been indicated that selection for AMEn is linked to an improved feed conversion ratio (**FCR**; Mignon-Grasteau et al., 2004) and a reduced environmental impact (de Verdal et al., 2011).

This relationship between digestibility and FE in broilers suggests that digestibility records have potential to aid in FE estimation. Literature on this relationship in laying hens is limited, but there are some indications for a similar trend (Ege et al., 2019; Van Hoeck et al., 2021). For example, Ege et al. (2019) observed that the digestion of nutrients for a fine ground diet was improved compared to a coarsely ground diet, and the FCR was lower for the hens fed the fine ground diet. To examine the relationship between digestibility and FE in more detail in laying hens, nutrient digestibility records of nutrient contents in manure of laying hens are required. Chemical analysis can be used to determine nutrients and subsequently calculate digestibility parameters: it is an analytical chemistry approach that uses classical methods (e.g., colorimetry, gravimetry and titration) to analyze nutritional values in samples and that can be used on faecal samples in laying hens to predict nutritional parameters (Lázaro et al., 2003). This suggests that there is potential to record digestibility components from manure in laying hens. These digestibility components can be used to calculate digestibility coefficients (**DCs**).

This study examines whether digestibility parameters, as determined from chemical analysis of laying hen manure, can serve as a predictor for FE in laying hens. To this end, two laying hen lines differing in FE (low versus high) were studied. The objective of this study was two-fold: to investigate 1) the variation in DCs between and within the two different lines, and 2) the use of DCs for prediction of FE in laying hens. The results of this study contribute to a better understanding of the relationship between DCs and FE and may aid in FE prediction in laying hens.

## Material and methods

### Birds and housing

Performance data, as well as manure and feed samples, were provided by Hendrix Genetics (Boxmeer, the Netherlands). In total, 100 laying hens from two purebred White Leghorn layer lines that differed in FE (lines A and B; 50 birds of each) were used in this study. Both lines were White Leghorn populations that were, independent from each other, selected for laying persistency of first quality eggs. The laying hens were kept at the R&D farm of Hendrix Genetics in Landhorst (the Netherlands), and for breeding purposes the birds were housed individually. The birds were housed in a battery system with 3 levels of individual cages. Individual records were collected over a period of 8 days, when the birds were between 90-96 weeks of age, at the end of the laying period. All hens were fed the same diet, a corn - wheat based diet including soybean meal and sunflower meal. The diet contained 2624 kcal (WPSA AME poultry), 16.0 % crude protein, 4.8 % crude fat, 4.5 % crude fiber, and 13.1 % crude ash. Resulting in 900 g/kg dry matter (**DM**), 25 g/kg nitrogen (**N**), 50 g/kg fat, 38 g/kg sugar and 131 g/kg ash. Feed samples were collected on three days (Table 1) and were frozen and stored in the freezer at -20°Celsius. Water was provided *ad libitum*.

**Table 1:** Overview of the individual data collection across the 8-day trial. The grey area indicates weekend days on which no data were collected.

Day in study	1	2	3	4	5	6	7	8
Manure (M)					M1, M2	M3, M4	M5, M6	M7
Feed sample (FS)				FS1		FS2		FS3
Egg (E)					E1^1^	E2	E3	E4
Feed weighing (FW)	FW1^2^			FW2	FW3	FW4	FW5	FW6
Body weight (BW)	BW1			BW2				BW3

1 Includes eggs from the three (potentially four) previous days

2 Feed weighed to determine the baseline for later feed consumption calculation.

### Individual records

During the sampling period performance data of the laying hens were collected as well, including daily feed intake, individual egg weight, number of eggs, and body weight (**BW**). From these records, egg mass (**EM**), daily body weight (**DBW**) and daily feed consumed (**DFC**) were determined (see Table 2 for abbreviations and trait descriptions). The laying percentage (**LP**) was calculated as the number of eggs laid divided by the expected number of eggs based on the laying period, multiplied by 100. The laying period that was included ranged from day 1 to 8. However, it could happen that eggs from the day before the data collection started (i.e., day 0) were also included in the collection and therefore the expected number of eggs was set to 8. To gain insight into FE in laying hens, two indicators were used: FCR (expressed as kilograms of feed consumed per kilogram of egg produced (Eq. 1)) and residual feed consumption (**RFC**; in grams; Eq. 2 from Luiting & Urff (1991)).

(1) $$FCR\;=\;\left(\frac{\text{sum}\;\text{of}\;\text{DFC}}{\text{EM}}\right)/7$$

(2) $$RFC\;=\;DFC-\left(b_{0}+b_{1}DBW+\;b_{2}EM+\;b_{3}BWG\right)$$

where FCR is the feed conversion ratio, DFC is the daily feed consumed in grams, EM is egg mass in grams which was calculated as the average EM multiplied with the laying percentage,7 is the period (in days) for which feed consumption was measured, RFC is the residual feed consumption in grams (i.e., the difference between the predicted and observed feed intake), DBW is the daily body weight in grams, and BWG is body weight gain in grams. In the calculation of RFC, the body weight gain was assumed to be negligible and thus set to zero, as we only studied a period of 7 days for adult birds. The implemented *b*-coefficients (not presented here for confidentiality considerations) were adjusted to account for the fact that the birds in this study were kept in a breeding environment, with a highly stable environmental temperature and lower levels of locomotion of the birds.

**Table 2:** Overview of the abbreviations and description of all the traits.

Abbreviation	Trait	Measurement unit
*Performance*		
DFC	daily feed consumed	g
EM	egg mass	g
DMW	daily manure weight	g
DBW	daily body weight	g
LP	laying percentage	%
*Digestibility*		
FCR	feed conversion ratio	
RFC	residual feed consumption	g
*Digestibility coefficients*		
DC_DM	digestibility coefficient for dry matter	%
DC_Fat	digestibility coefficient for fat hydrochloric	%
DC_N	digestibility coefficient for nitrogen	%
DC_Org	digestibility coefficient for organic matter	%
ADM	weighed after air-drying divided by the amount of manure before air-drying	g/kg
AMEn	apparent metabolizable energy	kcal/kg

### Manure sampling

Individual manure samples were collected two times per day (in the morning at 07.00 hours and afternoon at 15.00 hours), on days 5 to 8 of the study (see Table 1). On day 8, only in the morning a manure sample was collected, resulting in a total of 7 manure samples per bird in a period of 3.5 days (Table 1). The manure samples were weighed on a scale with a 1-gram accuracy to determine the daily manure weight (**DMW**). The manure samples were collected in freezer-zip-lock plastic bags. The samples were frozen at the location of sampling (below -4°Celsius) and were stored for maximum one day at the sampling location. Every evening all samples were transported and stored in a freezer at -80°Celsius. After the 3.5-day sampling period, manure samples were transported while frozen (below -4°Celsius) to the lab where they were stored in a freezer at -20°Celsius.

### Chemical analysis

The collected manure samples of the laying hens were analyzed by chemical analysis. For the chemical analysis, one pooled manure sample per animal for the whole period was used. For this, 20-25 % of each manure subsample from each bird was taken. This resulted in about 100 gram of the fresh manure sample per bird for the whole period. The remaining part of each subsample was immediately put back into the freezer. The manure samples were mixed, air dried (at 70°C for 1-2 days), weighed after air-drying and ground at 1 mm. Air-dried manure (**ADM)** is the weighed manure after air-drying divided by the amount of manure before air-drying. Before the chemical analysis was performed, the manure samples were stored at room temperature. Using chemical analysis, DM, ash, N, Fat hydrochloric (**Fat**) and uric acid (**UA**) were determined (all in g/kg). DM was measured gravimetrically after drying at 103°C (ISO 6496). Ash was measured gravimetrically after incineration at 550°C (ISO 5984). N was measured by the Dumas method (ISO 16634-1). Fat hydrochloric acid was measured gravimetrically after hydrolysis with hydrochloric acid followed by extraction with petroleum ether (ISO 6492). UA was measured spectrometrically after extraction with lithium carbonate followed by a specific colour reaction using a test kit (Human, Germany).

### Digestibility coefficients

Digestibility coefficients are a measure of the proportion of nutrients which are taken up from the digestive tract and therefore are not excreted in the manure. Lower DC values indicate that less nutrients are taken up from the digestive tract (and more excreted in manure) compared to higher DC values. The chemical components that were determined in the air-dried manure samples were used as a starting point for DC determination. These components were converted to levels for fresh manure (using the amount of dry matter in the fresh sample as the conversion factor), because the DMW was also calculated in the fresh manure samples. The DC for DM (**DC_DM**) was calculated based on the ratio of the amount of manure and amount of feed using Eq. (3).

(3) $$DC\_DM=100-\left(\frac{amount\;of\;manure}{amount\;of\;feed}*100\right)$$

where the amount of manure (g DM) was calculated according to Eq. (4) and the amount of feed (g DM) was calculated according to Eq. (5).

(4) $$\frac{ADM\;}{1000}*DMW$$

(5) $$DFC*\frac{DM\;in\;feed\;}{1000}$$

where ADM was the content of dry matter in the manure (g/kg), DMW was the daily manure weight (g), DFC was the daily feed consumption (g), and DM was the content of dry matter in the feed (g/kg). The DC for fat (**DC_Fat**), nitrogen corrected for uric acid (**DC_N**) and organic matter (**DC_Org**) were calculated according to Eq. (6).

(6) $$DC_{Nutrient}=100-\left(\frac{amount\;of\;manure\;\times \;content\;Nutrient\;in\;manure}{amount\;of\;feed\;\times \;content\;Nutrient\;in\;feed}\times \;100\right)$$

Where the contents of a nutrient in the manure or feed are expressed in g/kg DM, Nutrient was either Fat, N or Org and the amount of manure (g DM) was calculated using Eq. (4). The content of N in the manure was corrected for the content of N bound in uric acid. More detailed information on the correction of nitrogen in manure and the separately specified formulas used to calculate the DCs can be found in **Supplementary Information S1**.

### Statistical analysis

The analyses were carried out in R (R Core Team, 2019). For the overall descriptive statistics, histograms and boxplots were made for the different traits. Outliers were removed based on boxplots using the interquartile range as threshold, resulting in 43 measurements for line A and 44 measurements for line B remaining for further analyses. The chemical components were all normally distributed. The values for FCR, RFC and all DCs deviated from a normal distribution but applying a log transformation did not improve the distribution. Therefore, untransformed data were used. Differences in performance and DCs between line A and line B were examined using t-tests. A principal component analysis (**PCA**) was performed using the prcomp function, to summarize the variation of the different performance traits and DCs and to visualize the coherence between them. Linear models were used for predicting FCR or RFC based on 1) performance traits or 2) DCs. First, it was tested which effects to include in the full model. DFC and laying percentage were not included in the full model, because these two traits were confounded with FE, which is calculated based on DFC and egg mass (indirectly dependent on laying percentage). Moreover, the DCs were strongly correlated (see also results section) and therefore only one DC (DC_DM (the most straightforward to record in practice), DC_N or DC_Org) was included at a time in the DC models. Finally, this resulted in the following two linear models per line, for performance traits and DCs respectively:

(7) $$y=\mu +b_{1}*EM+b_{2}*DBW+e$$

where *y* was FCR or RFC, μwas the overall mean, $b_{1}\;$was the regression coefficient of EM on *y* with EM representing egg mass, $b_{2}\;$was the regression coefficient of DBW on *y* with DBW representing the daily body weight and e was the residual term, and

(8) $$y=\mu +b_{1}*DC+\;e$$

where *y* was FCR or RFC, μ was the overall mean, $b_{1}\;$was the regression coefficient of DC on *y* with DC representing the digestibility coefficient for dry matter, nitrogen, or organic matter and e was the residual term. To visualize the results, the packages Hmisc (Harrel, 2019) and corrplot (Wei and Simko, 2017) were used. The level of statistical significance was set at 0.05.

## Results

### Performance and digestibility differences between the two lines

Overall, there were significant differences in performance traits between the two lines, except for DBW (Table 3). The largest proportional difference between the two lines was found for DMW (121.6 g versus 146.6 g for line A and B, respectively). Surprisingly, the DMW (146.6 g) was even higher than the DFC (122.8 g) in line B. Line A hens on average consumed 142 g of feed per day, which is significantly higher compared to the DFC of line B hens. Furthermore, EM and LP were significantly higher for line B hens (53.8 and 92.0 %, respectively) compared to line A hens (49.3 and 82.0 %, respectively). In terms of digestibility, line A hens showed a higher FCR and RFC than line B hens (3.0 versus 2.3 and 1364.8 versus 1153.5, respectively; Table 3).

**Table 3:** Mean values with standard error (SE) for the 43 laying hens of line A and 44 hens of line B. P-values are shown for between-line differences.

Trait^1^	Line A (SE)	Line B (SE)	p-value
*Performance*			
DFC (g)	142.0 (6.7)	122.8 (2.4)	0.008
EM (g)	49.3 (1.5)	53.8 (1.1)	0.015
DMW (g)	121.6 (3.6)	146.6 (3.5)	<0.001
DBW (g)	1718.0 (26.1)	1683.0 (20.9)	0.301
LP (%)	82.0 (2.0)	92.0 (1.6)	<0.001
*Digestibility*			
FCR	3.0 (0.2)	2.3 (0.1)	<0.001
RFC	1364.8 (65.0)	1153.5 (21.2)	0.016

1 DFC = daily feed consumed, EM = egg mass, DMW = daily manure weight, DBW = daily body weight, LP = laying percentage, FCR = feed conversion ratio, and RFC = residual feed consumption.

### Digestibility components

The lowest DC was found for DC_DM for which 74.6 % was digested in line A hens and 65.5 % in line B hens. All DCs were significantly higher in line A compared to line B (Table 4). The variation that was observed differed between traits, ranging from 0.9 % for DC_Fat and DC_Org to 1.3 % for DC_DM and DC_N in line A and from 0.5 % for DC_Org to 1.1 % for DC_N in line B, and between lines with a highest difference of 0.4 % for DC_DM.

**Table 4:** Means with standard errors (between brackets) of the digestibility coefficients calculated from the chemical analyses’ components of 43 laying hens of line A and 44 hens of line B. P-values are shown for between-line differences.

Trait^1^	Line A	Line B	p-value
DC_DM	74.6 (1.0)	65.5 (0.6)	<0.001
DC_Fat	86.0 (0.8)	78.8 (0.7)	<0.001
DC_N	77.5 (1.0)	73.5 (0.8)	0.002
DC_Org	77.5 (0.8)	69.8 (0.5)	<0.001

1 DC_DM = digestibility coefficient for dry matter, DC_Fat = digestibility coefficient for fat hydrochloric, DC_N = digestibility coefficient for nitrogen and DC_Org = digestibility coefficient for organic matter.

### Correlation performance traits and DC within lines

**Fig. 1:**
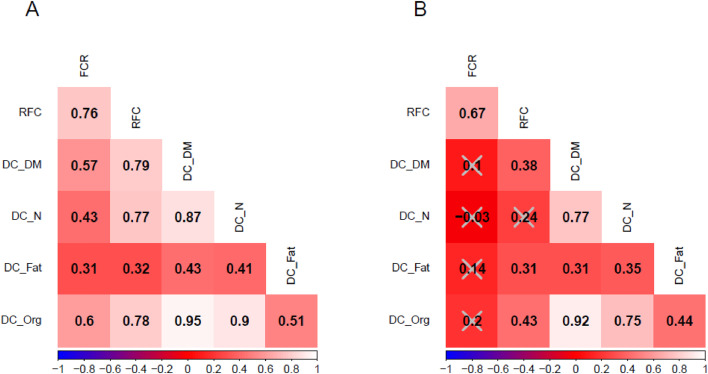
Correlations among the digestibility coefficients, FCR and RFC, with grey crosses representing correlations that were not statistically significant, for the 43 laying hens in line A (A) and the 44 laying hens in line B (B).

For line A, all correlations among DCs, RFC and FCR were significantly different from zero, moderate to high, and ranged from 0.31 (between FCR and DC_Fat) to 0.95 (between DC_DM and DC_Org) (Fig. 1**A**). Correlations of FCR or RFC with DCs ranged from 0.31 (between FCR and DC_Fat) to 0.79 (between RFC and DC_DM). In general, the correlations of RFC with DCs were stronger than the correlations of FCR with DCs (Fig. 1**A**). For line B, all correlations were significantly different from zero, except for the correlations between FCR and each DC and the correlation between DC_N and RFC (Fig. 1**B**). Correlations among FCR, RFC and DCs were, in general, lower in line B compared to line A. Significant correlations among DCs ranged from 0.31 (between DC_Fat and DC_DM) to 0.92 (between DC_DM and DC_Org) in line B. Significant correlations of RFC with DCs ranged from 0.31 (between RFC and DC_Fat) to 0.43 (between RFC and DC_Org) in line B.

### Principal component analysis

**Fig. 2:**
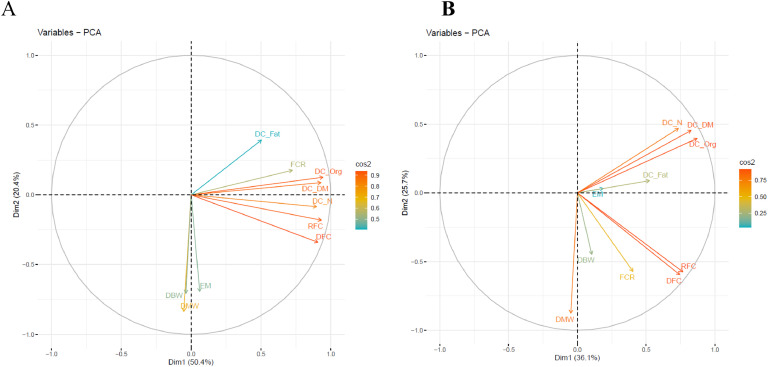
Graphical overview of the principal component analysis for the 43 laying hens in line A (A) and the 44 laying hens in line B (B).

The percentages of explained variance for the first and second dimension of the PCA analysis were 50.4 % and 20.4 %, respectively, in line A and 36.1 % and 25.7 %, respectively, in line B. The same general pattern of percentage of explained variance for the first two dimensions was seen for line A and line B (Fig. 2). DCs, except for DC_Fat, mostly contributed to the first dimension and the performance traits mostly contributed to the second dimension in both line A and B. DC_Org had the largest contribution in the first dimension and DMW in the second dimension, in both line A (Fig. 2**A**) and line B (Fig. 2**B**). In the third and fourth dimension, the percentage of explained variance decreased to 12.9 % and 7.6 %, respectively, for line A and 16.5 % and 10.8 %, respectively, for line B.

### Prediction models

The R^2^ values of the prediction models per line differed between FCR and RFC, as well as between performance traits or DCs as model predictors (Table 5). For FCR, the prediction model based on DC_Org resulted in the highest R^2^ (0.35) in line A, whereas the prediction model based on performance traits resulted in the highest R^2^ (0.47) in line B. For RFC, the prediction models based on DCs resulted in the highest R^2^ in both line A (ranging from 0.58 for DC_N to 0.62 for DC_DM) and B (ranging from 0.04 for DC_N to 0.17 for DC_Org). In the performance models, EM had a larger estimated effect on FCR than DBW, for both lines (Table 6). For FCR, the estimate for EM in line A was -0.06 ± 0.02 (*P* < 0.001), meaning that if the EM increased with 1 g, the predicted FCR decreased with 0.06. For line B, EM was also a significant predictor (estimate = -0.03 ± 0.00, *P* < 0.001; Table 6). The performance models for RFC showed no statistically significant contribution of EM. For the DC models, there was a large line difference. For line A, DC_DM, DC_N and DC_Org were shown to have a positive predicted effect on both FCR (estimate = 0.09 ± 0.02, *P* < 0.001, estimate = 0.07 ± 0.02, *P* < 0.01 and estimate = 0.12 ± 0.03, *P* < 0.001, respectively) and RFC (estimate = 50.16 ± 6.00, *P* < 0.001, estimate = 51.08 ± 6.62, *P* < 0.001 and estimate = 62.48 ± 7.70, *P* < 0.001, respectively). For line B, only DC_DM and DC_Org were shown to have a positive predicted effect on RFC (estimate = 13.23 ± 5.02, *P* < 0.05 and estimate = 18.36 ± 5.94, *P* < 0.01, respectively) whereas the other predictors in the DC model did not predict FCR or RFC well.

**Table 5:** R-squared (R^2^) of the models (for performance and digestibility coefficients) for line A and for line B.

Trait^1^	Model^2^	Line A	Line B
FCR	Performance	0.22	0.47
FCR	DC_DM	0.31	0.00
	DC_N	0.16	0.00
	DC_Org	0.35	0.02
RFC	Performance	0.00	0.00
RFC	DC_DM	0.62	0.12
	DC_N	0.58	0.04
	DC_Org	0.61	0.17

1 FCR = feed conversion ratio, and RFC = residual feed consumption

2 Performance model: $y=\;mu+b_{1}*DEW+b_{2}*DBW+e$ (EM is egg mass and DBW is daily body weight); Digestibility coefficients model: $y=\;mu+b_{1}*DC+\;e$ (DC is DC for dry matter, nitrogen or organic matter).

**Table 6:** Results of the linear regression models for the relationship between FCR or RFC with performance or digestibility coefficients for line A and line B.

Line	Trait^1^	Model^2^	Coefficients	Estimate	SE	t-value	Pr(>|t|)
A	FCR	Performance	Intercept	4.74	1.57	3.02	<0.01
			EM	−0.06	0.02	−3.76	<0.001
			DBW	0.00	0.00	0.77	0.44
		DC_DM	Intercept	−4.01	1.57	−2.55	<0.05
			DC_DM	0.09	0.02	4.47	<0.001
		DC_N	Intercept	−2.72	1.89	−1.44	0.16
			DC_N	0.07	0.02	3.03	<0.01
		DC_Org	Intercept	−6.64	2.00	−3.33	<0.01
			DC_Org	0.12	0.03	4.83	<0.001
	RFC	Performance	Intercept	1181.88	701.43	1.69	<0.1
			EM	4.43	7.11	0.62	0.54
			DBW	−0.02	0.40	−0.05	0.96
		DC_DM	Intercept	−2379.56	449.82	−5.29	<0.001
			DC_DM	50.16	6.00	8.36	<0.001
		DC_N	Intercept	−2595.38	514.49	−5.05	<0.001
			DC_N	51.08	6.62	7.72	<0.001
		DC_Org	Intercept	−3474.67	597.94	−5.81	<0.001
			DC_Org	62.48	7.70	8.11	<0.001
B	FCR	Performance	Intercept	2.78	0.53	5.23	<0.001
			EM	−0.03	0.01	−5.95	<0.001
			DBW	0.00	0.00	2.64	<0.05
		DC_DM	Intercept	1.75	0.88	1.98	<0.1
			DC_DM	0.01	0.01	0.64	0.53
		DC_N	Intercept	2.47	0.74	3.34	<0.01
			DC_N	0.00	0.01	−0.22	0.83
		DC_Org	Intercept	0.83	1.12	0.74	0.46
			DC_Org	0.02	0.02	1.32	0.20
	RFC	Performance	Intercept	855.75	296.00	2.89	<0.01
			EM	1.65	3.00	0.55	0.59
			DBW	0.12	0.16	0.79	0.43
		DC_DM	Intercept	286.66	329.61	0.87	0.39
			DC_DM	13.23	5.02	2.64	<0.05
		DC_N	Intercept	685.63	288.19	2.38	<0.05
			DC_N	6.37	3.91	1.63	0.11
		DC_Org	Intercept	−127.91	415.38	−0.31	0.76
			DC_Org	18.36	5.94	3.09	<0.01

1 FCR = feed conversion ratio, and RFC = residual feed consumption

2 Performance model: $y=\;mu+b_{1}*DEW+b_{2}*DBW+e$ (EM is egg mass and DBW is daily body weight); Digestibility coefficients model: $y=\;mu+b_{1}*DC+e$ (DC is DC for dry matter or nitrogen or organic matter).

## Discussion

In animal production, digestibility of nutrients is an essential factor in the evaluation of diets (Zaefarian et al., 2021). Feed composition and its characteristics are important factors contributing to the variability of digestibility. In this study, the first objective was to investigate the variation in digestibility coefficients within and between two different layer lines. The second objective was to investigate whether DC can aid in prediction of FE in laying hens. This study showed that there were significant differences in performance traits, FCR and RFC between line A and B. Moreover, several DCs were significantly different between line A and B. However, line A with higher DCs also showed a higher FCR and RFC, which contrasts with our expectations. The results for line B were not the same as for line A. There appears to be potential for FE prediction based on DCs, although this seems to be line-dependent, making across-line prediction challenging.

The two laying hen lines used in this study were chosen based on their *a priori* known differences in FE. Differences between the lines were clearly visible in this study, with line A showing a higher DFC, FCR and RFC than line B (Table 3). Surprisingly, hens from line B produced more manure (in g) than feed was consumed (g). One explanation might be that the manure in line B contained a higher proportion of water, as the fresh manure weight was used here and the amount of DM in manure was 38.1 (g) for line B (and 30.7 g for line A), which was indeed lower than the amount of feed consumed. However, Rahman et al. (2012) reported that different laying hen strains show differences in feed consumption, FE, manure excretion and manure moisture content, but not in dry matter manure production. This contrasts with the observation in the current study that ADM was also observed to be numerically higher in line B (260.2 g/kg) compared to line A (252.4 g/kg; results not shown). However, De Verdal et al. (2011) studied two broiler lines that were divergently selected on AMEn and observed that the low AMEn line had both a higher fresh excreta weight and a higher dry excreta weight. Moreover, it has been observed that divergent selection on AMEn has resulted in changes in the gastrointestinal tract (De Verdal et al., 2010) and that several excretion traits, such as fresh excreta weight relative to feed intake, are heritable (De Verdal et al., 2011). This suggests that there might be differences in digestive efficiency due to genetics, which could explain the differences observed between the laying hen lines in this study as well. Moreover, it is important to note that hens from line A likely had higher feed waste levels, as it was observed, but not quantified, that the laying hens from line A played more with their feed compared to line B. This suggests that the amount of feed consumed by the hens from line A might be overestimated, which could explain the large difference in DFC and the yet small difference in DEW between line A and B.

The differences in performance between line A and B are contradictory to the differences in digestibility between the lines; hens from line B were more efficient in terms of FCR and RFC, whereas these hens had lower digestibility coefficients than line A for all components. Based on observations in broilers (Ten Doeschate et al., 1993), it was expected that the birds with the more efficient feed conversion would also show higher DCs. This unexpected result might be partly caused by the possible overestimation of DFC for line A, resulting in an increased calculated FCR. Furthermore, the relationship between DCs and FCR in poultry is not a one-on-one relationship, as FCR is related to many other traits, such as temperature and hydrogen sulfide concentrations in poultry houses (Kocaman et al., 2006) and genetics (Yuan et al., 2017). Moreover, laying hens divergently selected on FCR and housed individually show behavioral differences, in terms of frequency of walking, resting, cage pecking, drinking, preening, and time spent feeding (Clark et al., 2019). In addition, in broilers it was shown that much of the observed variation in FE was due to age and body weight differences (Chambers and Lin, 1988). This highlights that digestibility alone might not explain the observed differences in FCR. However, in the current study the ages and body weights of the laying hens were similar, as well as their housing environment. However, the hens used in this study were relatively old (90-96 weeks). It might be that line A has a slightly different aging process than line B, and that subsequently their physiological age might differ. Another possible explanation for the contradictory results in terms of efficiency and digestibility is that both lines received the same feed, but the specific feed requirements and the utilization of the feed components may have differed between the lines. In broiler lines selected for digestion efficiency, line by diet interactions in starch and protein digestibility have been observed (Rougiére et al., 2009). Another explanation might be that the hens in both lines differ in post absorptive metabolism; some hens are better at converting nutrients to egg than others. Gu et al. (2021) showed that age could reduce the intestinal barrier function and leads to lower digestive enzyme activities, nutrient retention, and egg quality after the peak laying stage. This indicates that nutritional interventions might not work as well in late-phase laying hens compared to younger hens.

The PCA results showed that DC_Org had the largest contribution in the first dimension and DMW in the second dimension, in both lines A and B. This suggest that DC_Org and DMW are key traits to record for laying hens. The large contribution of DC_Org in the first dimension is very interesting, since this is a new trait which might be used to predict FE. The prediction models also indicated other traits of interest for prediction of FCR and RFC. In terms of performance, increases in EM were shown to be linked to a lower predicted FCR in both lines. Given the calculation of FCR that includes EM as a component, this makes sense: if the DFC would remain unchanged but the EM would increase, this would result in a lower FCR. Besides the relationships between FE and performance traits, the results of this study also showed that DC are related to FE in laying hens, but mainly in line A and not in line B. In line A, DC_DM was shown to have a predicted positive effect on both FCR and RFC, while in line B this was not the case. Studies in broilers have indicated that there are correlations between individual digestibilities and characteristics of the gastrointestinal tract (Maisonnier et al., 2001). For example, the apparent digestibility of amino acids is positively correlated with the ratio of gizzard weight over body weight and the duodenum weight-length ratio (Maisonnier et al., 2001). Given the earlier-mentioned changes in the gastrointestinal tract as a result of divergent selection on AMEn (De Verdal et al., 2010), it is possible that the two lines here also differ in their gastrointestinal tract (e.g., length, mass, or enzymes), even though the lines were not specifically selected on FE. Consequently, different DC may play roles to a different extent in relation to FE in these lines. More research is needed to investigate this, for example also examining the gastrointestinal tract of these hens in more detail. Overall, it is important to keep in mind that FE prediction based on DC may not generalize well across poultry lines, and that therefore FE prediction requires additional investigation in the poultry line of interest before implementation.

## Conclusions

In this research, it was studied whether digestibility coefficients, as determined from chemical analysis of manure, can serve as a predictor for feed efficiency in two laying hen lines. Differences in performance and DCs were observed between the two laying hen lines. Correlations between DCs and FCR were observed, indicating that DCs can aid in prediction of FE in laying hens. However, the contribution of different DCs in the prediction of FE differed between the two lines, making across-line FE prediction challenging in laying hens. For practice, this line-dependency limits the generalisability of the results from one line to another. Nevertheless, the DC data show potential for FE prediction, and, depending on costs and effort, it may be valuable for breeding companies to have line-specific prediction models. Overall, the data on DCs collected in this study contribute to an improved understanding of differences in digestibility between laying hen lines.

## CRediT authorship contribution statement

**Ghyslaine C.B. Schopen:** Writing – review & editing, Writing – original draft, Visualization, Formal analysis. **Marco C.A.M. Bink:** Writing – review & editing, Methodology, Conceptualization. **Estella Leentfaar:** Writing – review & editing, Methodology, Conceptualization. **Dirkjan Schokker:** Writing – review & editing, Methodology, Conceptualization. **Malou van der Sluis:** Writing – review & editing, Writing – original draft, Visualization, Formal analysis. **Leon H. de Jonge:** Formal analysis, Writing – review & editing. **Lisanne M.G. Verschuren:** Writing – review & editing, Methodology. **Carmen Jansen-Noordijk:** Writing – review & editing, Data curation. **Esther D. Ellen:** Writing – review & editing, Supervision, Project administration, Methodology, Funding acquisition, Conceptualization.

## Disclosures

The authors declare that they have no known competing financial interests or personal relationships that could have appeared to influence the work reported in this paper.

## Appendix Supplementary materials

Image, application 1
