# Supplementary material for: Variation in digestibility parameters related to feed efficiency between and within two laying hen lines
Source: Poult Sci. 2025 Aug 23;104(11):105719. doi: 10.1016/j.psj.2025.105719 (PMC12433497; doi:10.1016/j.psj.2025.105719)
Supplement: Supplementary file 1 [file mmc1.docx]

**Supplementary Information S1: Formulas to calculate digestibility coefficients.**

**DC_Fat**

DC_Fat = $100-\left( \frac{amount of manure * content Fat in manure}{amount of feed * content Fat in feed}*100 \right)$

where the amount of manure (g DM) * content Fat in manure (g/kg DM) was calculated as

$$\left( \frac{ADM}{1000}*DMW \right)*\left( \frac{Fat in manure* \frac{1000}{\mathrm{DMman}}}{1000} \right)$$

and the amount of feed (g DM) * content Fat in feed (g/kg DM) was calculated as

$$DFC*\frac{Fat in feed}{1000}$$

where ADM was the content of dry matter in the manure (g/kg), DMW was the daily manure weight (g), Fat was the content of fat (g/kg), DMman was the content of dry matter in manure (g/kg) and DFC was the daily feed consumption (g).

**DC_N**

DC_N = $100-\left( \frac{amount of manure * content N in manure}{amount of feed *content N in feed}*100 \right)$

where the amount of manure (g DM) * content N in manure (g/kg DM) was calculated as

$\left( \frac{ADM}{1000}*DMW \right)*\left( \frac{N in manure* \frac{1000}{\mathrm{DMman}}}{1000} \right)$

and the amount of feed (g DM) * content N in feed (g/kg DM) was calculated as

$$DFC*\frac{N in feed}{1000}$$

where ADM was the content of dry matter in the manure (g/kg), DMW was the daily manure weight (g), N in manure was the content of nitrogen in manure (g/kg) corrected for uricacid (N in manure = N content – 1/3 * uricacid content), DMman was the content of dry matter in manure (g/kg) and DFC was the daily feed consumption (g).

**DC_Org**

DC_Org = $100-\left( \frac{amount of manure * content Org in manure}{amount of feed * content Org in feed}*100 \right)$

where the amount of manure (g DM) * content Org in manure (g/kg DM) was calculated as

$\left( \frac{ADM}{1000}*DMW \right)*\left( \frac{\left( DMman -Ashman \right)* \frac{1000}{\mathrm{DMman}}}{1000} \right)$

and the amount of feed (g DM) * content Org in feed (g/kg DM) was calculated as

$$DFC*\frac{DM in feed-ash in feed}{1000}$$

where ADM was the content of dry matter in the manure (g/kg), DMW was the daily manure weight (g), Org was the content of organic matter (g/kg), DMman was the content of dry matter in manure (g/kg), Ashman was the content of ash in manure (g/kg) and DFC was the daily feed consumption (g).
